# Beyond One-Variable-at-a-Time Subgroup Analyses: Illustrating the Predictive Approaches to Treatment Effect Heterogeneity Framework in the Third International Stroke Trial

**DOI:** 10.1213/ANE.0000000000007783

**Published:** 2025-11-03

**Authors:** Markus Huber, Christian Beilstein, Patrick Y. Wuethrich, Thomas Vetsch

**Affiliations:** From the Department of Anaesthesiology and Pain Medicine, Inselspital, Bern University Hospital, University of Bern, Bern, Switzerland.

Heterogeneous treatment effects (HTEs) refer to non-random variations in treatment effects among individuals in both magnitude and direction. The assessment of HTEs may empower clinicians to individualize treatment decisions according to specific patient characteristics.^1^ In randomized controlled trials (RCTs), HTEs are traditionally examined with subgroup analyses, although the limitations of such analyses—ranging from multiplicity of comparisons, spurious findings, and low power of the interaction analyses—are well established.^2^

The past decades have seen major advances to overcome the clinical and statistical limitations of these so-called one-variable-at-a-time subgroup analyses, culminating in the recommendations of the Predictive Approaches to Treatment effect Heterogeneity (PATH) statement.^3^ In essence, the PATH framework describes 2 approaches to examine HTEs in RCTs by considering multiple variables per patient: The risk score approach and the effect score approach.^4^

The risk score approach groups patients according to their predicted risks of experiencing the outcome of interest rather than by levels of a single variable (eg, age category). The basic idea is then to examine possible variations of the treatment effect in the intervention and control group in these risk groups. Thus, the risk score approach considers risk-based variations in treatment benefit.^3^ The effect score approach attempts to model the effect of a particular intervention compared to the alternative treatment (eg, placebo) on a patient level, for example, by including the interaction of the treatment variable with a potential relative effect modifier (eg, age) in a regression model. Here, we focus on the risk score approach and illustrate its clinical relevance using a real-world RCT.

## METHODS

The Third International Stroke Trial (IST-3) was a multi-center, placebo-controlled randomized trial of intravenous thrombolytic therapy of the drug Alteplase (rt-PA) for patients with acute ischemic stroke during the years 2000–2011.^5^ The data are publicly available.^6^ For ethical research considerations (eg, with respect to the approval by the appropriate Institutional Review Board and written informed consent), we refer to the primary publication of the IST-3 trial.^5^

We examined possible HTEs for the primary binary outcome of the IST-3 trial: the proportion of patients alive and independent as measured by the Oxford Handicap Score with scores 0–2 at 6 months follow-up. Outlined in the PATH statement, the following steps were involved in our predictive HTE approach (note that risk here refers to a favorable outcome and the terms risk and probability are used interchangeably):

1. A risk score model for the primary outcome. Here, we computed a multivariable logistic regression-based risk prediction model for the primary outcome using directly the baseline variables of the IST-3 trial. Importantly, the model does not feature the allocated treatment as a covariate—it is “treatment-blinded” (Table).
2. A statistical test if the treatment effect of Alteplase versus placebo varies across the predicted baseline risks: The presence of HTEs was determined with a likelihood ratio test of the interaction between treatment and the linear predictor, where the latter was extracted from the multivariable logistic regression of step (1). Note that the likelihood ratio test involved a second logistic regression model with only treatment and the linear predictor (and their interaction) as covariates.
3. Separation (“binning”) of the predicted probabilities of a favorable outcome into quartiles and grouping of patients according to these risk quartiles (Q1–Q4).
4. Evaluation of the treatment benefit in the risk-based subgroups Q1–Q4. The treatment benefit was evaluated on the absolute risk difference scale.

**Table.** Summary Statistics of Selected Baseline Variables Collected at Randomization of the IST-3 Randomized Controlled Trial

	Complete case N = 3010	Multivariable logistic regression model
		Odds ratios
Outcome at 6-mo follow-up		
Alive and independent as measured by the Oxford Handicap Score (0–2)		
Yes	1074 (35.7%; 95% CI, 34.0–37.4)	
No	1936 (64.3%; 95% CI, 62.6–66.0)	
Predictors		
Treatment		
Placebo	1509 (50.1%)	Not included
Alteplase (rt-PA)	1501 (49.9%)	
Age (y)	81.0 [72.0; 86.0]	0.96; 95% CI, 0.95–0.97; *P* < .001
Sex		
Female	1562 (51.9%)	
Male	1448 (48.1%)	0.98; 95% CI, 0.80–1.20; *P* = .8
Lived alone before stroke?		
Yes	1125 (37.4%)	
No	1885 (62.6%)	0.90; 95% CI, 0.74–1.09; *P* = .3
Recent ischemic change likely cause of this stroke?		
No	1772 (58.9%)	
Possibly yes	699 (23.2%)	0.98; 95% CI, 0.79–1.23; *P* = .9
Definitely yes	539 (17.9%)	0.77; 95% CI, 0.58–1.00; *P* = .053
Received antiplatelet drugs in last 48 h?		
Yes	1550 (51.5%)	
No	1460 (48.5%)	1.03; 95% CI, 0.85–1.23; *P* = .8
Patient in atrial fibrillation at randomization?		
Yes	908 (30.2%)	
No	2102 (69.8%)	1.27; 95% CI, 1.02–1.57; *P* = .035
Systolic BP (mm Hg)	155.0 [140.0; 170.0]	1.00; 95% CI, 1.00–1.00; *P* > .9
Diastolic BP (mm Hg)	80.0 [72.0; 91.0]	1.00; 95% CI, 0.99–1.00; *P* = .2
Estimated weight (kg)	70.0 [62.0; 80.0]	1.01; 95% CI, 1.00–1.01; *P* = .035
Best eye response (Glasgow Coma Scale)		
Nonspontaneously (never, to pain, to command)	443 (14.7%)	
Spontaneously	2567 (85.3%)	1.98; 95% CI, 1.22–3.24; *P* = .006
Best motor response (Glasgow Coma Scale)		
Normal	2581 (85.7%)	
Not normal (none, extend to pain, abnormal flex to pain, normal flex to pain, localizes movements to pain)	429 (14.3%)	1.00; 95% CI, 0.58–1.73; *P* > .9
Best verbal response (Glasgow Coma Scale)		
None	373 (12.4%)	
Noises only	339 (11.3%)	0.86; 95% CI, 0.49–1.52; *P* = .6
Inappropriate words	317 (10.5%)	1.64; 95% CI, 0.80–3.30; *P* = .2
Confused in time, place, or person	383 (12.7%)	1.02; 95% CI, 0.40–2.49; *P* > .9
Orientated in time, place, and person	1598 (53.1%)	1.41; 95% CI, 0.44–4.22; *P* = .6
Total Glasgow Coma Scale score	14.0 [12.0; 15.0]	0.86; 95% CI, 0.67–1.12; *P* = .2
Total NIH Stroke Scale score	11.0 [6.0; 17.0]	0.83; 95% CI, 0.81–0.85; *P* < .001
Stroke subtype		
LACI	328 (10.9%)	
PACI	1137 (37.8%)	1.01; 95% CI, 0.76–1.34; *P* > .9
POCI	245 (8.1%)	1.11; 95% CI, 0.76–1.61; *P* = .6
TACI	1300 (43.2%)	0.82; 95% CI, 0.59–1.16; *P* = .3
Model performance		
*c* Statistic		0.83
Brier score		0.16
Likelihood ratio test for interaction (treatment × linear predictor)		0.0003

The 2 treatment groups were a placebo group and a treatment group receiving Alteplase (rt-PA). Categorical variables are shown with counts and frequencies, whereas numerical variables are summarized with medians and interquartile ranges. The associated odds ratios of a multivariable logistic regression model (not including the treatment variable) for the primary outcome of the IST-3 trial—the proportion of patients alive and independent as measured by the Oxford Handicap Score 0–2 at 6-mo follow-up—are illustrated with means and 95% CI. Model performance is measured by the *c* statistic and the Brier score. The statistical significance of the interaction between treatment and the linear predictor of the multivariable logistic regression model was assessed with a likelihood ratio test.

Abbreviations: 95% CI, 95% confidence interval; BP, blood pressure; IST-3, The Third International Stroke Trial; LACI, lacunar infarct; NIH, National Institutes of Health; PACI, partial anterior circulation infarct; POCI, posterior circulation infarct; rt-PA, recombinant tissue plasminogen activator; TACI, total anterior circulation infarct.

All analyses were performed with R and the code can be found on github: https://github.com/marbhuber/predictive_hte_ist3/.^7^

## RESULTS

Our complete-case analysis features N = 3010 patients of the original N = 3035 patients and N = 1074 (35.7%; 95% confidence interval [CI], 34.0–37.4) patients had a favorable outcome. The Table further shows the associated odds ratios of the multivariable prediction model, illustrating, for example, a better outcome for younger patients and patients with a lower Total National Institutes of Health Stroke Scale score. The model is well calibrated with a moderate discriminatory performance (*c* statistic of 0.83).

The Figure A compares the distributions of the predicted probabilities of a favorable outcome for the quartile-based risk groups and for 2 traditional one-variable-at-a-time subgroups (age category and sex). There is a large overlap in the probability distributions of the sex and age subgroups. In contrast, the quartile-based risk groups that incorporate information from multiple variables provide a more refined risk stratification. The overall distribution of the predicted probabilities of a favorable outcome is illustrated in the Figure B. The distribution is slightly skewed, showing that only a few patients with a high probability of a functional recovery account for a large number of favorable outcomes; the average probability is thus slightly higher than the median probability for a typical patient.

**Figure.**
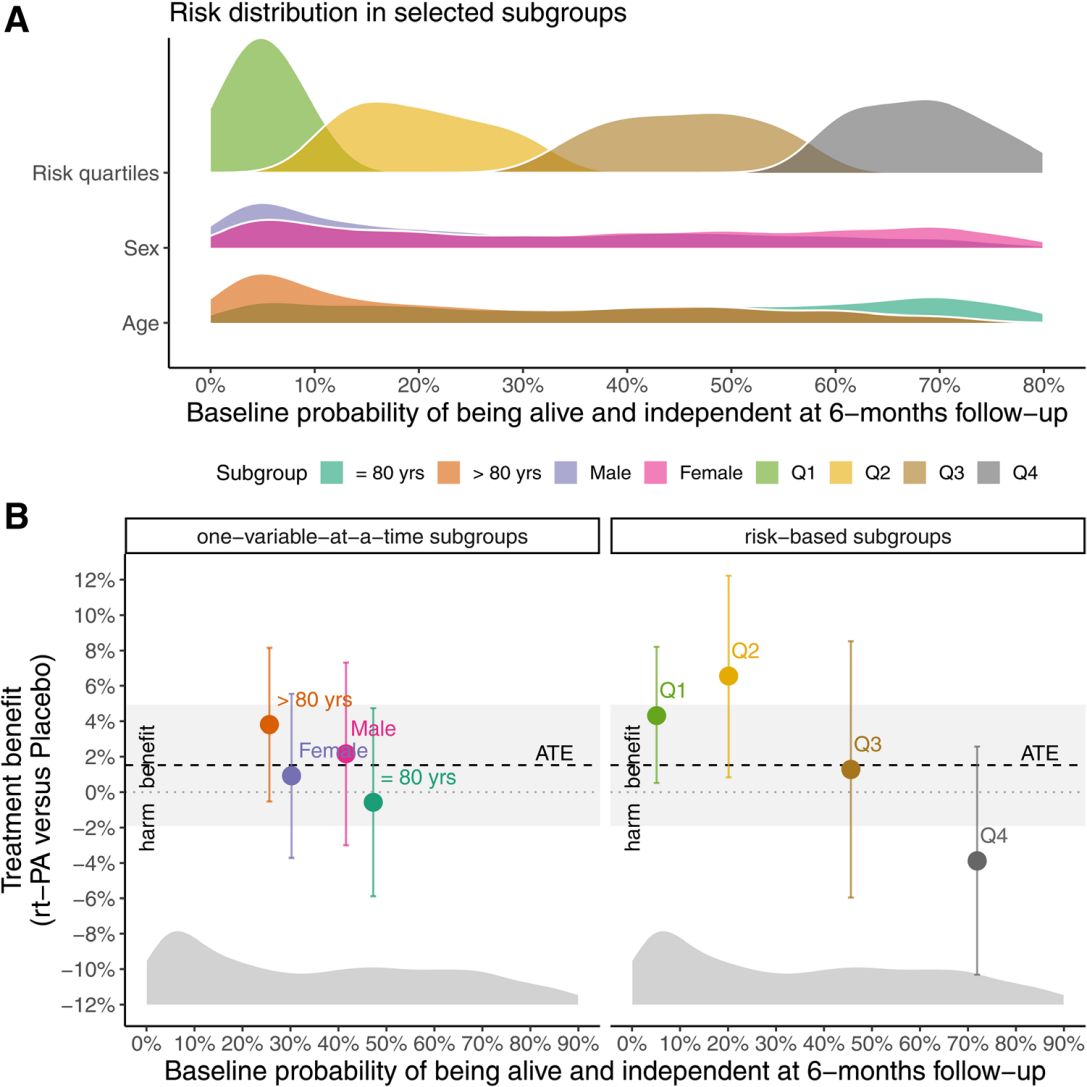
Illustration of a predictive approach to treatment effect heterogeneity. A, Distributions of the predicted probability of a favorable outcome (alive and independent as measured by the Oxford Handicap Score 0–2 at 6-mo follow-up) for quartile-based risk groups as predicted with a multivariable logistic regression model using a number of selected baseline variables collected at randomization (Table) and for 2 traditional one-variable-at-a-time subgroups (for age and sex). In this so-called ridgeline plot, the values of the probability densities are omitted for illustration purposes. For a particular subgroup, for example, patients in the lowest quartile-based risk group (Q1), the probability distribution’s peak refers to the most likely value of the predicted baseline probability of the primary outcome. The width of a subgroup’s probability distribution illustrates how much this predicted baseline probability can vary for patients in this subgroup. B, Treatment benefit of an Alteplase (rt-PA) treatment compared with placebo expressed on the absolute risk difference scale for traditional subgroups for age and sex (left-hand side) and for quartile-based risk groups (right-hand side). The solid dots show the mean treatment effect at the average baseline probability fo being alive and independent at 6-mo follow-up for each subgroup. Vertical error bars show the associated 95% confidence intervals. The distribution of the predicted probabilities of a favorable outcome is shown at the bottom of the figure. The estimate of the ATE and its 95% confidence interval are shown horizontally with a black dashed line and the gray ribbon. The gray dotted line denotes the null value of no treatment effect. ATE indicates average treatment effect; rt-PA, recombinant tissue plasminogen activator.

We found no overall treatment benefit with the intervention (risk difference of −1.5%; 95% CI, −4.9 to 1.9; *P* = .38) with respect to the primary outcome (Figure B). However, this masks significant treatment effect heterogeneity (*P* = .0003): while the treatment with Alteplase might be harmful for patients who have a high baseline probability of functional recovery (Q4), the drug may provide a benefit for patients with a low baseline probability of functional recovery (Q1 and Q2). The Figure B highlights that the 4 quartile-based risk groups span both a broader range of baseline probabilities of a favorable outcome and a broader range of subgroup-specific treatment benefits than the traditional one-variable-at-a-subgroups: the corresponding subgroups compare patients that do not differ substantially from the average risk—in contrast to the risk-based groups.^8^

## DISCUSSION

In conclusion, the fact that the overall treatment effect of a RCT may not apply to every patient has been firmly established over the past decades.^9,10^ The PATH statement provides a modern framework to look beyond the summary results of RCTs and there are several advantages of these predictive approaches to HTE compared to traditional subgroup analyses. Notably, these approaches specifically account for the fact that risk variables often cluster in patients and a risk-based evaluation of treatment benefits provides a more nuanced perspective on trade-offs of the benefits and harms of treatment than the overall trial result.^8^

This short analysis features the limitation that we could not describe in full detail the advantages and limitations of predictive approaches to HTE, for example, a detailed comparison between the risk modeling and the effect modeling approach. This would be beyond the scope of this Research Letter and for more details, the reader is referred directly to the PATH statement. We limited our analysis to the former approach due to the familiarity of many clinicians with clinical prediction modeling. Our analysis considered trial data to predict baseline risks—a so-called internal model. While valid, the PATH statement suggests to use a high-quality, externally developed model when available.^3^ We further considered quartiles to stratify the patients’ baseline risks. Note that the examination of the statistical significance of treatment effect heterogeneity does not depend on the choice of quantiles as the full linear predictor is considered (step 2). Quantiles or tertiles are often a sensible choice—increasing the number of risk groups would result in fewer patients per risk group and in larger confidence intervals.

In addition, the PATH statement provides detailed guidance with respect to applying a risk modeling approach to HTE in trials when there is no overall treatment effect.^3^ In the case studied here, one could argue that substantial heterogeneity in the IST-3 trial population could be anticipated and the treatment with Alteplase could potentially be harmful, thus warranting a predictive HTE analysis according to the PATH statement. We note that the primary publication of the IST-3 trial features a risk modeling approach to evaluate treatment effect heterogeneity.^5^ The overall objective of our study here is to motivate the use of a predictive approach to HTE by contrasting traditional one-variable-at-a-time subgroups with risk-based subgroups on the one hand and to illustrate the different steps involved in a predictive HTE analysis according to the PATH statement on the other hand.

## DISCLOSURES

**Conflicts of Interest:** None. **Funding**: This work was solely supported by departmental or institutional funding. **This manuscript was handled by:** Thomas R. Vetter, MD, MPH, MFA.
